# Supplementary material for: Lipid Serum Profiling of Boar-Tainted and Untainted Pigs Using GC×GC–TOFMS: An Exploratory Study
Source: Metabolites. 2022 Nov 15;12(11):1111. doi: 10.3390/metabo12111111 (PMC9693814; doi:10.3390/metabo12111111)
Supplement: Supplementary file 1 [file metabolites-12-01111-s001.zip › metabolites-2026315-supplementary.pdf]

Supplementary Materials

Lipid Serum Profiling of Boar-Tainted and Untainted Pigs Using GC_GC–TOFMS: An Exploratory Study

Kinjal Bhatt [1,*] , Thibaut Dejong [1], Lena M. Dubois [1], Alice Markey [2] , Nicolas Gengler [2] , José Wavreille [3], Pierre-Hugues Stefanuto [1] and Jean-François Focant [1]

Contents

Figure S1. Concentration range of lipids in human plasma as per NIST CoA.

Figure S2. Optimization plot of derivatization condition.

Figure S3. (a) Contour plot of FAMEs standard mixture (b) Zoomed-in contour plot of FAMEs standard mixture.

Figure S4. (a) Contour plot of NIST SRM 1950 standard (b) Zoomed-in contour plot of NIST SRM 1950 standard.

Figure S5. (a) Contour plot of pig serum (b) Zoomed-in contour plot of pig serum.

Table S1. Fatty acid composition (% Area) of boar tainted (BT, n = 20) and untainted (UT, n =20) pig serum

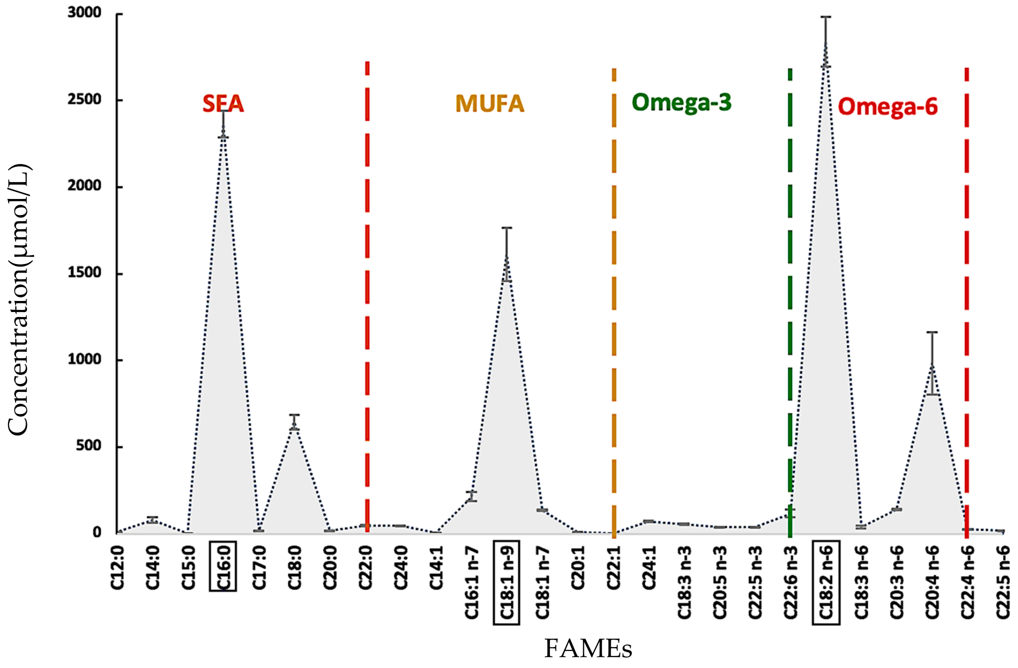

Figure S1. Concentration range of lipids in human plasma as per NIST CoA.

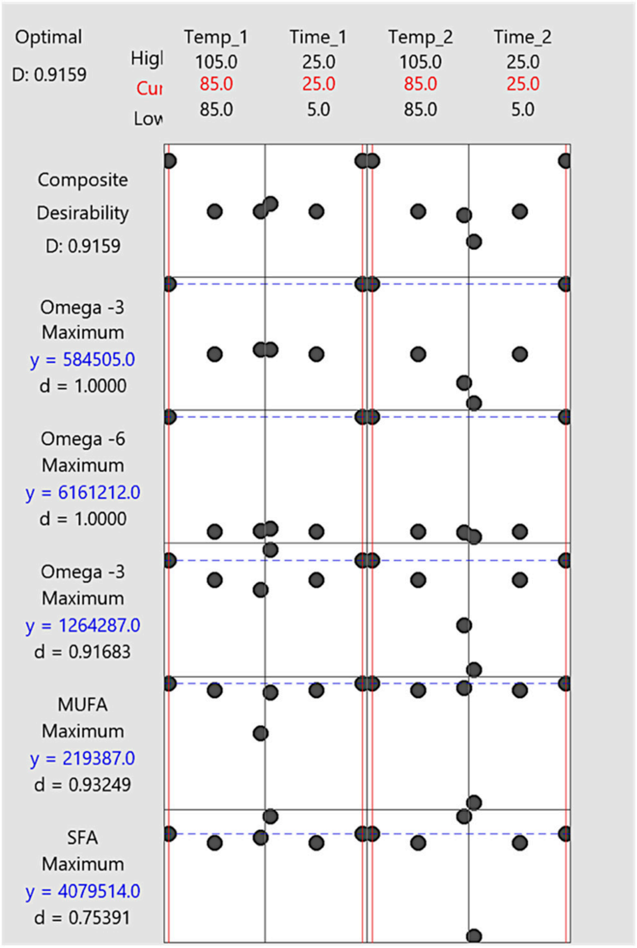

Figure S2. Optimization plot of derivatization condition.

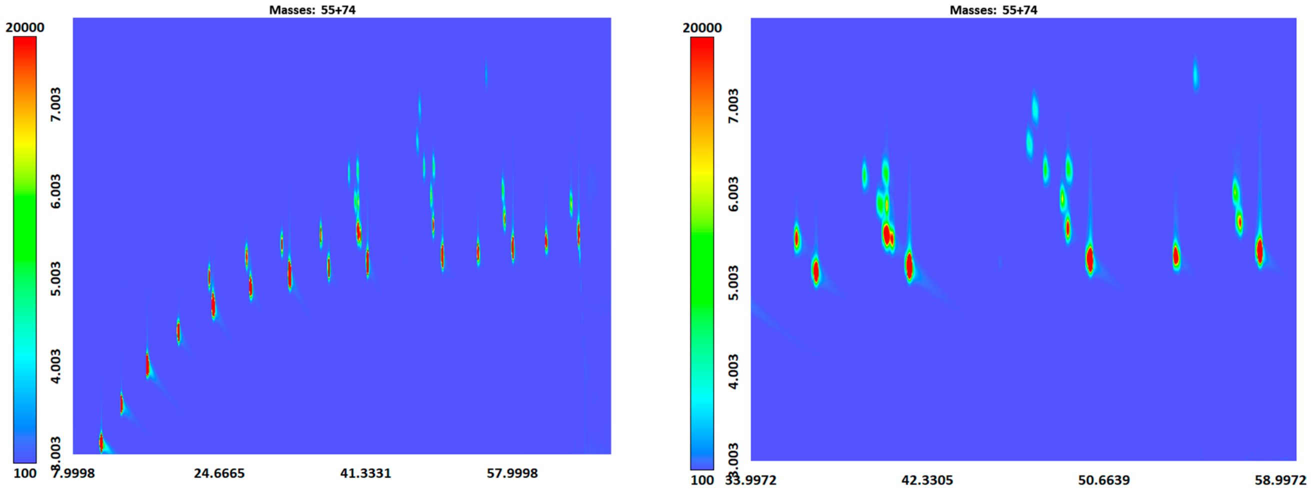

Figure S3. (a) Contour plot of FAMEs standard mixture (b) Zoomed in contour plot of FAMEs standard mixture.

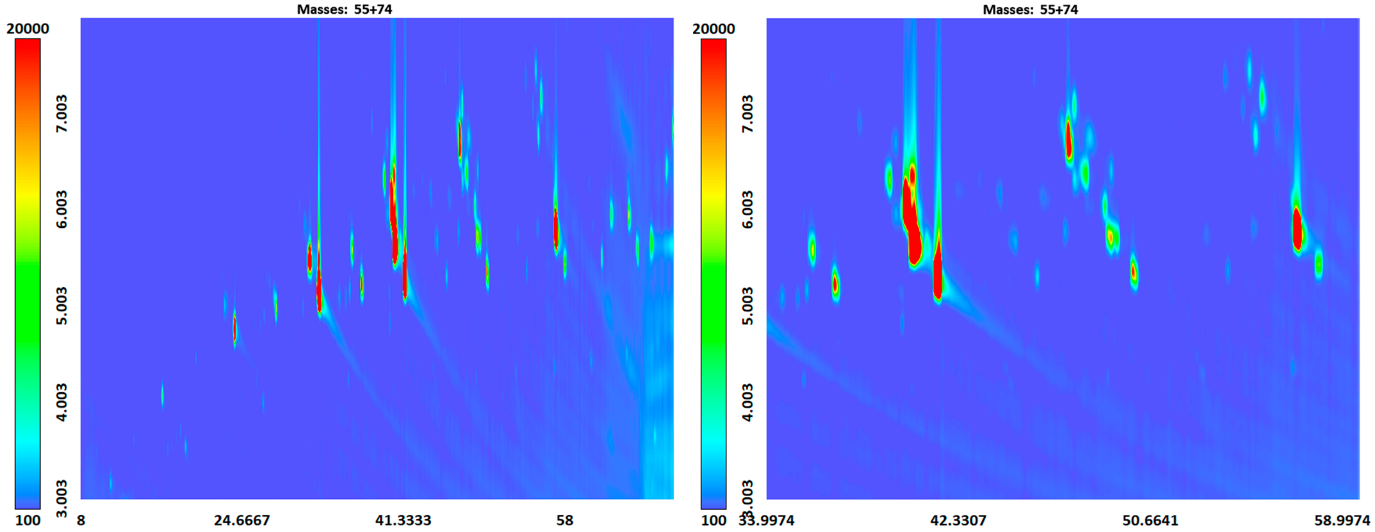

Figure S4. (a) Contour plot of NIST SRM 1950 standard (b) Zoomed in contour plot of NIST SRM 1950 standard.

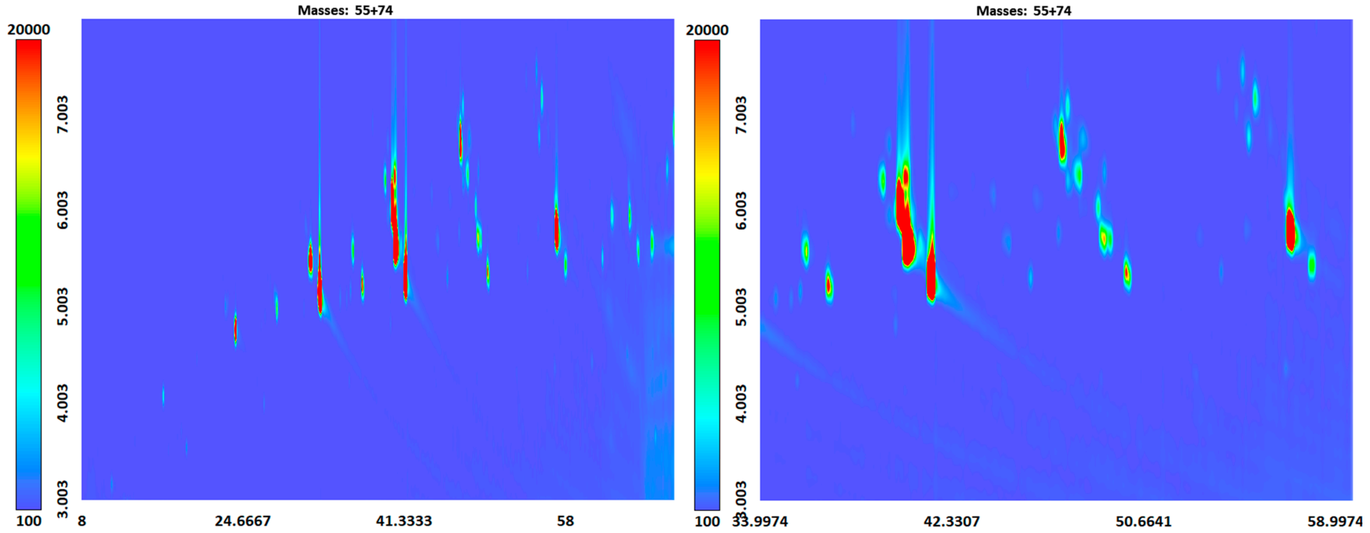

Figure S5. (a) Contour plot of pig serum (b) Zoomed in contour plot of pig serum.

Table S1. Fatty acid composition (% Area) of boar tainted (BT, n = 20) and untainted (UT, n =20) pig serum

	Name	Abbreviation	Class	Area% BT	Area% UT
1	Nonanoic acid, methyl ester	C9:0	SFA	0.09	0.07
2	Decanoic acid, methyl ester	C10:0	SFA	0.70	0.31
3	Dodecanoic acid, methyl ester	C12:0	SFA	0.20	0.09
4	Tetradecanoic acid, methyl ester	C14:0	SFA	1.36	0.39
5	Pentadecanoic acid, methyl ester	C15:0	SFA	0.56	0.22
6	Hexadecanoic acid, methyl ester	C16:0	SFA	20.35	14.30
7	Heptadecanoic acid, methyl ester	C17:0	SFA	0.63	0.30
8	Octadecanoic acid, methyl ester	C18:0	SFA	10.11	7.87
9	Nonadecanoic acid, methyl ester	C19:0	SFA	0.20	0.07
10	Eicosanoic acid, methyl ester	C20:0	SFA	0.57	0.61
11	Docosanoic acid, methyl ester	C22:0	SFA	0.67	0.39
12	Tricosanoic acid, methyl ester	C23:0	SFA	0.38	0.29
13	Tetracosanoic acid, methyl ester	C24:0	SFA	1.48	0.65
			**ΣSFA**	37.29	25.56
14	7-Hexadecenoic acid, methyl ester	C16:1 n-9	MUFA	2.55	2.10
15	10-Heptadecenoic acid, methyl ester	C17:1 n-7	MUFA	0.81	1.01
16	13-Octadecenoic acid, methyl ester	C18:1 n-5	MUFA	10.26	9.65
17	10-Nonadecenoic acid, methyl ester	C19:1 n-9	MUFA	0.03	0.04
18	9-Eicosanoic acid, methyl ester	C20:1 n-9	MUFA	0.09	0.08
19	11-Eicosanoic acid, methyl ester	C20:1 n-11	MUFA	1.81	1.31
20	13-Docosenoic acid, methyl ester	C22:1 n-9	MUFA	8.62	6.52
21	15-Tetracosenoic acid, methyl ester	C24:1	MUFA	0.08	0.08

			**ΣMUFA**	24.24	20.78
22	9,12,15-Octadecatrienoic acid, methyl ester	C18:3 n-3	PUFA, (ω-3)	2.59	1.55
23	6,9,12,15 Octadecatetraenoic acid, methyl ester	C18:4 n-3	PUFA, (ω-3)	1.74	0.95
24	11,14,17-Eicosatrienoic acid, methyl ester	C20:3 n-3	PUFA, (ω-3)	1.42	0.67
25	8,11,14,17-Eicosatetraenoic acid, methyl ester	C20:4 n-3	PUFA, (ω-3)	0.60	0.31
26	5,8,11,14,17-Eicosapentaenoic acid, methyl ester	C20:5 n-3	PUFA, (ω-3)	0.21	0.15
27	7,10,13,16,19-Docosapentaenoic Acid (DPA)	C22:5 n-3	PUFA, (ω-3)	0.41	0.28
28	4,7,10,13,16,19-Docosahexaenoic acid, methyl ester (DHA)	C22:6 n-3	PUFA, (ω-3)	0.77	0.28
			**ΣPUFA, (ω-3)**	7.75	4.19
29	9,12-Octadecadienoic acid, methyl ester	C18:2 n-6	PUFA, (ω-6)	15.60	21.06
30	6,9,12-Octadecatrienoic acid, methyl ester	C18:3 n-6	PUFA, (ω-6)	0.25	0.87
31	11,14-Eicosadienoic acid, methyl ester	C20:2 n-6	PUFA, (ω-6)	0.10	0.19
32	8,11,14-Eicosatrienoic acid, methyl ester	C20:3 n-6	PUFA, (ω-6)	0.63	1.12
33	5,8,11,14 -Eicosatetraenoic acid, methyl ester	C20:4 n-6	PUFA, (ω-6)	0.24	0.44
34	13,16-Docasadienoic acid, methyl ester	C22:2 n-6	PUFA, (ω-6)	0.42	3.23
35	7,10,13,16-Docosatetraenoic acid, methyl ester	C22:4 n-6	PUFA, (ω-6)	2.75	5.10
36	4,7,10,13,16- Docosapentaenoic acid, methyl ester	C22:5 n-6	PUFA, (ω-6)	0.05	0.14
37	5,8,11-Eicosatrienoic acid, methyl ester	C20:3 n-9	PUFA, (ω-9)	0.68	0.52
			**ΣPUFA (ω-6), (ω-9)**	20.72	32.68
38	Cholesta-3,5-diene	Cholesterol derivatives_1	Cholesterol dvt	7.33	11.00
39	Cholesta-2,4-diene	Cholesterol derivatives_2	Cholesterol dvt	2.67	5.79
			**ΣCholesterol dvt**	10.00	16.79
